# Statistical Learning Across Development: Flexible Yet Constrained

**DOI:** 10.3389/fpsyg.2012.00598

**Published:** 2013-01-11

**Authors:** Lauren Krogh, Haley A. Vlach, Scott P. Johnson

**Affiliations:** ^1^Department of Psychology, University of CaliforniaLos Angeles, CA, USA; ^2^Department of Educational Psychology, University of WisconsinMadison, WI, USA

**Keywords:** infants, auditory statistical learning, visual statistical learning, language acquistion, learning constraints, statistical learning mechanisms

## Abstract

Much research in the past two decades has documented infants’ and adults’ ability to extract statistical regularities from auditory input. Importantly, recent research has extended these findings to the visual domain, demonstrating learners’ sensitivity to statistical patterns within visual arrays and sequences of shapes. In this review we discuss both auditory and visual statistical learning to elucidate both the generality of and constraints on statistical learning. The review first outlines the major findings of the statistical learning literature with infants, followed by discussion of statistical learning across domains, modalities, and development. The second part of this review considers constraints on statistical learning. The discussion focuses on two categories of constraint: constraints on the types of input over which statistical learning operates and constraints based on the state of the learner. The review concludes with a discussion of possible mechanisms underlying statistical learning.

## Introduction

To survive, an organism must be capable of organizing and interpreting the constant stream of sensory input it receives. Research in the last two decades has revealed powerful statistical learning abilities in infants and adults, including the developing capacity to extract statistical regularities from a variety of auditory inputs including artificial and natural language (e.g., Saffran et al., 1996a; Saffran et al., 1996b; Pelucchi et al., 2009) and non-linguistic auditory stimuli (Saffran et al., 1999). An independent line of research has extended these findings to the visual domain, demonstrating infants’ and adults’ sensitivity to statistical patterns within visual arrays and sequences of shapes (e.g., Fiser and Aslin, 2001, 2002a,b; Kirkham et al., 2002, 2007; Bulf et al., 2011).

The current review discusses auditory and visual statistical learning to elucidate both its generality and its constraints. We first outline the major findings of the statistical learning literature with infants, followed by discussion of statistical learning across domains, modalities, and development. The second part of this review considers constraints on statistical learning. The discussion focuses on two categories of constraint: constraints on the types of input over which statistical learning operates, and constraints based on the state of the learner. The review concludes with a discussion of possible mechanisms underlying statistical learning.

## Auditory Statistical Learning

### Artificial language

Given the richness and complexity of a natural language, how is it that infants acquire vocabulary and structure so rapidly, and seemingly effortlessly, in their first years after birth? For example, one challenge facing young language learners is the fact that speakers do not mark word boundaries with pauses, and listeners must rely on other information to accomplish this task. Early in the “cognitive revolution,” researchers hypothesized that the statistical structure of language might be important for word segmentation (Harris, 1955; Hayes and Clark, 1970). For instance, Hayes and Clark (1970) tested adults’ ability to segment “words” from a continuous stream of speech analogs in which the only cue to word boundaries was the distribution of the phonemes. Adult participants successfully segmented words, suggesting sensitivity to statistical information in speech. However, Hayes and Clark did not specify a mechanism to account for this result.

Building upon these findings, Saffran et al. (1996a,b) proposed a mechanism for statistical word segmentation: transitional probability (TP) detection. In their experiments, adults, first-graders, and 8-month-olds were presented with a continuous stream of speech from an artificial language in which word boundaries were indicated by differing TPs between syllables within words (high TPs) and across word boundaries (low TPs). After brief exposure to this language, listeners in all three age groups were able to distinguish between high TP syllable sequences (“words”) and low TP sequences (“part-words”). Thus, both infant and adult learners appeared sensitive to the TP information contained in the speech stream, suggesting that statistical learning via sensitivity to TPs is a possible mechanism contributing to language acquisition.

Although such early studies in infant statistical learning conceptualized statistical learning as sensitivity to a particular conditional relation, TP, more recent research highlights a variety of other conditional statistics (e.g., mutual information) that could be used to distinguish words from foil items. This point is discussed in greater detail in a subsequent section, however we mention it briefly here to point out that, although several studies are described in terms of differing TPs, it remains unclear which conditional relations participants rely upon to segment sequences.

One limitation to the design of the aforementioned studies was that frequency information co-varied with conditional probability statistics. That is, high TP words occurred more frequently than low TP part-words in the learning (familiarization) phase of the experiment, and it remained unclear whether participants distinguished syllable sequences based on differences in conditional relations or simply differential frequencies of occurrence during learning. To address this issue, Aslin et al. (1998) conducted a “frequency-balanced” version of their original study, with words and part-words appearing equally frequently, such that only sensitivity to conditional relations could be used to distinguish the two types of sequences. Aslin et al. found that 8-month-old infants were still able to distinguish high and low TP sequences. This result suggests that infants can track conditional probability information independent of co-occurrence frequency and use this information to determine word boundaries. Taken together, this work demonstrated the potential for statistical learning to support early language acquisition.

The possibility that statistical learning is a primary mechanism underlying early language acquisition raises the question of the age at which statistical learning is functional in young infants. Teinonen et al. (2009) examined statistical learning in sleeping newborns by presenting a continuous stream of three-syllable words in an artificial language similar to that employed by Saffran et al. (1996a), in which the only cues to word boundaries were the conditional relations or frequencies of co-occurrence between syllables. Using electroencephalography, they measured newborns’ event-related potential (ERP) negativities to the first, second, and third syllables in the words. Teinonen et al. (2009) found a significant difference between the ERP negativity to the first and third syllables, indicating that the neonatal brain is sensitive to word boundaries marked by conditional relations and reacts differently during word onset compared to word offset. This research demonstrates, therefore, that statistical learning is functional even in newborn infants, and perhaps contributes to language acquisition even prior to birth.

For statistical learning to be a primary mechanism underpinning infants’ early language acquisition, however, it must be able to scale up to the demands of more complex natural language (Johnson and Tyler, 2010). The aforementioned studies employed artificial speech composed entirely of bisyllabic words or entirely of trisyllabic words. Natural language, in contrast, consists of much more varied word types. To simulate more natural language learning, Johnson and Tyler (2010) investigated infants’ ability to segment an artificial language composed of both bi- and trisyllabic words. Interestingly, neither 5.5- nor 8-month-old infants were able to segment this language, suggesting that certain characteristics of natural language, such as varied word length, may make segmentation more difficult compared to segmentation of artificial languages.

Other research, however, suggests that some characteristics of natural language may help to make statistical word segmentation possible. For instance, Thiessen et al. (2005) found that 7-month-olds were able to segment an artificial language containing words of varying length when the language was produced with infant- but not adult-directed prosody. As an artificial language becomes more complex (here, by consisting of words of mixed, as opposed to uniform, length), therefore, other natural speech cues such as exaggerated prosody may be needed to facilitate statistical word segmentation.

Indeed, conditional probabilities have never been posited as the sole cue to word segmentation in natural language. Instead, researchers have suggested that initial sensitivity to conditional probabilities may facilitate language acquisition by bootstrapping sensitivity to other linguistic cues. For instance, in English, lexical stress serves as a cue to word boundaries as a majority of English words are stressed on their first syllable (Thiessen and Saffran, 2003). Statistical segmentation mechanisms may facilitate sensitivity to stress cues by providing infants with an inventory of words from which they can discover the dominant stress pattern of their native language (Thiessen and Saffran, 2003, 2007; Swingley, 2005).

In the next section, we discuss research that provides even stronger support for the possibility that statistical learning contributes to language acquisition by examining infants’ statistical learning in natural language.

### Natural language

The aforementioned research focused on statistical learning in the context of synthesized artificial languages. More recent research has examined more natural language learning contexts, such as sequences of grammatically correct and semantically meaningful sentences in natural speech. Pelucchi et al. (2009) examined 8-month-olds’ ability to extract statistical regularities from an unfamiliar natural language (Italian for English-learning infants). Infants were presented with a constant stream of fluent infant-directed Italian speech for approximately 2 min. After this brief exposure, infants provided evidence of discrimination between high- and low-TP bisyllabic words. Importantly, both types of words had occurred equally frequently in the speech stream, indicating that infants were using conditional probability information, not simply frequency information, in discriminating between words.

The Pelucchi et al. (2009) results imply that infants discriminated likely from unlikely sound sequences in natural language, but they leave open the critical question of how learners represent extracted statistical information. Saffran (2001) took an important step in addressing this question by asking whether English-learning infants treat segmented syllable sequences as candidate English words or simply as highly probable sound sequences. In this experiment, 8-month-old infants were familiarized to a continuous stream of artificial speech composed of nonsense words similar to those used in Saffran et al. (1996a). Following familiarization to the stimuli, infants participated in a post-familiarization test. This test compared infants’ listening time to speech in which words and part-words were embedded in either simple English (e.g., “I like my *tubido*”) or matched nonsense (e.g., “zy fike ny *tubido*”) frames. If infants treated the outputs of statistical learning simply as highly probable sound sequences, both the English and nonsense frame conditions should have elicited similar listening preferences. However, if infants treated the outputs of statistical learning as candidate English words, then they should have shown differential listening preferences when those units were embedded in English versus nonsense frames. Saffran found that infants exposed to English frames listened significantly longer to words in this English context than to part-words, and that this difference in listening preference for words versus part-words did not extend to the nonsense frame condition. These results suggest that the statistical learning mechanisms underlying word segmentation do generate word-like units and raises the question of whether these units are available to support other aspects of language acquisition, such as mapping words to meaning.

Establishing a link between sound and meaning is an essential aspect of language acquisition, particularly for young language learners. Graf Estes et al. (2007) investigated the connection between statistical word segmentation and object-label learning in 17-month-olds. Infants were presented with 2.5 min of fluent speech composed of bisyllabic nonsense words where the only cues to word boundaries were the conditional relations between syllables. Immediately following this segmentation task, infants were habituated to two object-label combinations, presented one at a time. For each combination, infants heard a bisyllabic sound sequence from the segmentation task while viewing a 3D object on a computer screen. For half the infants, the bisyllabic sound sequences were words from the segmentation task, and for the other half, the sound sequences were non-words (Experiment 1) or part-words (Experiment 2). Following habituation to these two object-label pairings, infants were presented with two types of test trials. “Same” test trials presented the same object-label combinations from the habituation phase. “Switch” test trials switched the labels for the two objects such that the label for object 1 was played while the infant viewed object 2. Longer looking on switch trials would suggest that infants were sensitive to the change in word-object pairings and was therefore taken as evidence of acquisition of the object-label associations. Graf Estes et al. found that only infants exposed to words from the segmentation task as object labels looked longer on switch compared to same test trials. This indicates that by 17 months of age, infants may be able to map newly segmented sound sequences (“words”) to novel objects as linguistic labels, but are unable to do so with non-words or part-words. These results support the claim that statistically segmented sound sequences are word-like and suggest that the output of auditory statistical learning is represented linguistically.

Recent work has also found associations between statistical learning abilities and natural language processing (Conway et al., 2010; Misyak and Christiansen, 2012). For instance, Misyak and Christiansen (2012) found that even after controlling for measures of short-term and working memory, vocabulary, reading experience, cognitive motivation, and fluid intelligence, performance on statistical learning tasks was the key predictor of comprehension of natural language sentences. Such findings suggest that statistical learning may be relevant to language learning not only because extracted statistical information may be represented linguistically, but also because statistical and language learning might overlap in their underlying mechanisms (Christiansen et al., 2007; Misyak and Christiansen, 2012; see also work on cross-situational statistical learning, e.g., Smith and Yu, 2008).

### Non-linguistic stimuli

Demonstrations that conditional probability information extracted from auditory input is represented linguistically (Saffran, 2001; Graf Estes et al., 2007) and that learners form associations between auditory statistical learning and language learning (Conway et al., 2010; Misyak and Christiansen, 2012) raise the question whether statistical learning is language-specific, or whether it also operates over non-linguistic stimuli. In the auditory domain, Saffran et al. (1999) found that both infants and adults appeared to detect statistical regularities in non-linguistic sequences of “tone words.” The procedure and stimuli used were modeled directly after those used in Saffran et al.’s (1996a,b) studies employing speech, allowing for a direct comparison of participants’ performance with tones and syllables. Both adults and infants performed with similar accuracy in discriminating words from part-words, regardless of whether these units were instantiated in syllables or tones. These findings suggest that statistical structure can be extracted from auditory input regardless of the domain in which it is presented (syllables or tones), and raise the possibility that statistical learning might also function over input from other modalities.

## Visual Statistical Learning

Investigating infants’ and adults’ extraction of statistical structure in visual input addresses the question of domain-generality by asking whether or not statistical learning is limited to auditory input.

### Infants

Kirkham et al. (2002) examined infants’ detection of statistical regularities from sequentially presented visual information. Two-, 5- and 8-month-old infants were habituated to a continuous stream of six looming colored shapes presented one at a time with no breaks or pauses between shapes. The six shapes were organized into three pairs that were presented in random order such that the boundaries between pairs were defined by TPs (TP = 1.0 within pairs, TP = 0.33 between pairs). Following habituation, infants viewed six test displays alternating between the familiar habituation sequence and a novel sequence composed of the same six shapes from habituation presented in random order. Infants at all three ages exhibited a significant novelty preference, suggesting that the infants were sensitive to statistical regularities that defined the visual shape sequences. This was the first published experiment to demonstrate not only infants’ sensitivity to statistically defined structure in visual sequences, but also to suggest that statistical learning is a domain-general learning process, capable of identifying statistical structure across modalities.

The Kirkham et al. (2002) study was also the first to investigate the developmental time course of visual statistical learning during the first year after birth. Kirkham et al. found no significant differences in novelty preferences between age groups. This lack of observed development, combined with the finding that statistical structures could be detected after only a few minutes of exposure, suggests visual statistical learning may be functional at or soon after the onset of visual experience. Bulf et al. (2011) explored this possibility by investigating whether infants are capable of extracting statistical regularities from visual sequences at birth. Bulf et al. employed a habituation design similar to that used by Kirkham et al. (2002), presenting newborn infants (mean age 38 h) with continuous sequences of either four or six looming shapes following a statistically defined structure. Newborns provided evidence of detecting the structure of the shape sequences, though only in sequences composed of four, not six, shapes. Thus, statistical learning appears to be functional at birth, operating over both auditory (Teinonen et al., 2009), and visual input (Bulf et al., 2011), but is constrained, an issue we discuss in greater detail in a subsequent section.

The method of testing employed by Kirkham et al. (2002) and Bulf et al. (2011) demonstrated that infants can discriminate between structured and random sequences. However, it did not indicate what statistical or structural features allowed infants to make this discrimination. Rather than computing conditional statistics, as has been found in studies of auditory statistical learning, infants could have been responding to a variety of other features, such as frequency of shape co-occurrence, which co-varied with conditional probability information. Determining which features infants are sensitive to is important for understanding the extent and utility of statistical learning as detection of different statistical features allow varying degrees of associative learning and inference. For instance, *co-occurrence statistics* inform the observer of the likelihood of two events occurring together, but leave the observer uncertain of the likelihood of an event occurring given that the other has taken place. In contrast, *conditional probability statistics* serve to reduce uncertainty by measuring the predictive power of one event with respect to another. Reducing uncertainty contributes to efficient coding of sensory information and is thought to be essential for associative learning (see Fiser and Aslin, 2002b). Thus, a learning mechanism that allows detection of conditional probability statistics would support more effective learning, including the prediction of the likelihood of future events, relative to co-occurrence frequency.

Fiser and Aslin (2002a) examined whether infants were sensitive to conditional probability statistics in visual input in addition to co-occurrence frequency. They habituated 9-month-olds to looming multi-element scenes, then tested infants’ preference for various element pairs that had occurred in the scenes. The researchers found that infants preferred not only element pairs that co-occurred more frequently as embedded elements in scenes, but also pairs that had higher conditional probability (viz., predictability) between elements in the pair. Thus, infants were sensitive to the statistical coherence of the elements within visual scenes in addition to co-occurrence frequency. In sum, this research demonstrates infants’ sensitivity to conditional relations in both auditory and visual input, suggesting that statistical learning is a domain-general process. In the next section, we outline research with adults that provides even stronger support for this idea by examining statistical learning of more complex visual stimuli and the generalizability of statistical learning across contexts.

### Adults

Although research with infants has begun to demonstrate the robustness of statistical learning for detecting statistical structure in visual scenes and sequences, the complexity of the visual structures examined in infant studies are rather simplistic compared to those examined in studies with adults. For example, research with adults has examined learners’ sensitivity to first- as well as higher-order statistics, and has employed more complex multi-element scenes and sequences than those used with infants to examine the flexibility of the representations learners extract from such input.

Fiser and Aslin (2001) explored the range of first- and higher-order statistics that adults compute during passive viewing of visual scenes. Participants viewed a total of 12 shapes, which were divided into six base pairs. Three of these pairs appeared at a time in various positions within either a 3 × 3 or 5 × 5 grid “scene.” The relations between any two shapes in a scene could be described in terms of co-occurrence and conditional probabilities. Each base pair appeared in half of the scenes, such that the probability of co-occurrence of the two shapes in each of the six base pairs was 0.5. Because the two objects composing each base pair always occurred together within a scene, shapes within base pairs had a conditional probability of 1.0. Fiser and Aslin found that adults detected first-order statistics (single-shape frequency) as well as several higher-order statistics from the scenes. Specifically, participants detected absolute shape-position relations within the grid and shape-pair arrangements independent of grid position. Most importantly, even when the probabilities of co-occurrence of some base pairs and non-base pairs were equated, adults were still able to distinguish the familiar base pairs based solely on their (higher) conditional probabilities.

The finding that adults are capable of implicitly extracting higher-order statistics from static spatially presented visual stimuli led Fiser and Aslin (2002b) to probe this ability further with temporally presented stimuli. In this experiment, adult participants viewed 12 shapes organized into four temporal triplets, such that after the first element of the triplet appeared on the screen, the second and then the third elements of the triplet always followed. There were no pauses or breaks between successive shapes such that the triplet structure could only be learned via temporal-order statistics among pairs or triplets of shapes. Just as with spatially presented visual stimuli, participants became sensitive to first-order as well as higher-order statistics in the temporal shape sequences. Participants retained the frequency of individual shapes and distinguished sequences of shapes presented during familiarization from both novel sequences of familiar shapes and sequences of shapes seen during familiarization but presented less frequently. Interestingly, when frequency information and co-occurrence probabilities were equated, adults were still able to distinguish shape sequences based on differing conditional probabilities.

These demonstrations of visual statistical learning with both temporally and spatially presented input raises the question of how such information is represented and whether such representations might generalize to new contexts. Turk-Browne and Scholl (2009) demonstrated that learning of statistical regularities in temporal shape sequences (finding shape “triplets” in a continuous stream of shapes) was expressed in static spatial configurations of these same shape triplets. Similarly, learning of statistically defined spatial configurations (base pairs, as in Fiser and Aslin, 2001) facilitated detection performance in temporal streams (Turk-Browne and Scholl, 2009). Thus, visual statistical learning in adults appears to produce flexible representations that can be generalized to new situations. Such transferability is likely important for visual statistical learning to be practical in ever-changing real-world visual environments.

## Constraints on Statistical Learning

The generalizability of statistical learning across tasks and domains raises the important question of whether and what constraints may exist on statistical learning. If one considers the infinite number of possible statistical relations that could be computed at each level of representation, it becomes clear that for statistical learning to be feasible, it must be constrained. What are these constraints?

### Types of input

It is unlikely that all statistical regularities are learned equally well, given the infinite number of possible statistics that could be extracted from the environment. Rather, research suggests that statistical learning mechanisms preferentially track statistical regularities in the types of input that occur most frequently in the natural environment (Newport and Aslin, 2004; Conway and Christiansen, 2009; Emberson et al., 2011).

#### Spatial versus sequential input

Intuitively, there seem to be structured differences in the organization of auditory and visual information in the natural environment. For instance, auditory information is conveyed temporally whereas visual information is arrayed spatially. Moreover, each sensory modality seems to process particular aspects of environmental input. For instance, a brief snapshot is typically enough time to recognize a complex visual scene whereas at least several seconds are needed to recognize a voice or melody (Conway and Christiansen, 2009). These intuitions are supported by studies of perception and memory suggesting that spatial information weighs most prominently in visual cognition, whereas temporal information weighs most prominently in audition (see Conway and Christiansen, 2009 for a discussion). Such modality differences raise the question of whether statistical learning processes might be constrained to preferentially track statistics in input that accords with the auditory-temporal, visual-spatial structure of the environment.

Conway and colleagues (Conway and Christiansen, 2005, 2009; Emberson et al., 2011) examined how modality differences may constrain implicit statistical learning. For example, Conway and Christiansen (2009) investigated whether vision and audition exhibited different constraints on statistical learning of spatially and temporally structured information. Conway and Christiansen compared learning of one statistically defined structure presented in three different formats: auditory information presented temporally (pure tones of various frequencies presented one at a time through headphones), visual information presented temporally (different colored squares presented one at a time in the center of screen), and visual information presented spatially (the same colored squares presented simultaneously left to right in a horizontal row across the center of the screen). The task was an artificial grammar learning (AGL) task in which adult learners were presented with a set of training sequences that adhered to a specific rule-governed finite state grammar. After the learning task, learners were presented with a test on classifying novel sequences as being either legal (generated by the same rules as the training sequences) or illegal. The results demonstrated that participants in the visual-spatial condition classified test sequences with a similar degree of accuracy as participants in the auditory condition. However, participants in the visual-temporal condition were significantly less accurate in their classifications compared to those in the auditory condition. This ability to acquire the structure of spatially arrayed visual input as well as temporally structured auditory, but not visual, input suggests that adults’ statistical learning may be constrained to preferentially track statistics in inputs that accord with the auditory-temporal, visual-spatial structure of the environment.

#### Presentation rate

Of course, human learners, including young infants, provide evidence of detecting statistical patterns in sequential visual input under some circumstances (e.g., Fiser and Aslin, 2002b; Kirkham et al., 2002; Bulf et al., 2011). A recent study by Emberson et al. (2011) helped to reconcile these seemingly contradictory findings by investigating the mediating role of presentation timing in statistical learning of auditory and visual information. Their results suggest that there is an interaction of presentation format (spatial versus sequential) and presentation timing in constraining statistical learning across modalities.

Emberson et al. (2011) compared visual and auditory statistical learning in an interleaved familiarization design. Adult learners were presented with a visual stream of abstract shapes organized into triplets that was interleaved pseudo-randomly with an auditory stream of monosyllabic nonsense words also organized into triplets. Participants were randomly assigned to either attend to the visual stream or the auditory stream, and given a cover task (detecting repeat elements in only that stream) to ensure that attention was allocated to the appropriate stream. Following familiarization, participants were tested on learning in each modality. During test trials, participants judged which of two sequences seemed more familiar: a triplet from familiarization or a foil sequence that did not adhere to the triplet structure. Importantly, this study compared effects of variation in presentation rate. In the “fast” condition, elements were presented for 225 ms with an ISI of 150 ms, resulting in an SOA of 375 ms. In the “slow” condition, elements were presented with an SOA of 750 ms.

Emberson et al. (2011) found that performance in the unattended modality did not differ from chance in any condition. At the fast presentation rate, the statistical relations between adjacent elements were only learned in the attended *auditory* stream. At the slow presentation rate, the opposite effect occurred: only the relations between adjacent elements in the attended *visual* stream were learned. Emberson et al. posited that visual statistical learning improved with the slower rate of presentation because it was less temporally demanding on the visual system. In contrast, auditory statistical learning was impaired at the slower presentation rate because of weaker perceptual grouping cues. That is, when sequential elements were separated by longer intervals, they were less likely to form a single perceptual unit or stream, hindering the detection of statistical information in the stream. Taken together, these results document complex constraints on statistical learning that accord with the structure of the natural environment, with relatively rapid presentation of temporal information critical for auditory statistical learning, and either static spatial information or relatively slowly presented temporal information critical for visual statistical learning.

#### Natural language: types of non-adjacent regularities

This interaction of presentation format and timing in statistical learning illustrates one way in which constraints on the types of information over which statistical learning operates may reflect environmental structure. Some researchers have additionally argued that constraints on learning not only reflect, but also help to explain, structural aspects of the environment, such as those found in natural languages (e.g., Christiansen and Chater, 2008). For example, a wide range of adjacent regularities appear throughout natural languages, but the types of non-adjacent regularities languages exhibit are quite constrained.

Newport and Aslin (2004) investigated the intriguing possibility that constraints on the types of non-adjacent statistical computations that learners perform may match and even drive observed constraints on non-adjacent regularities in natural languages. For example, it is common for natural languages to contain non-adjacent regularities relating elements of one kind while skipping over intervening elements of a different kind. In Hebrew and Arabic, word stems are formed out of phonemic segments of one kind (consonants), while intervening segments are of another kind (vowels). In contrast, it is uncommon for natural languages to contain non-adjacent regularities in which intervening items are of the same kind as that in which the non-adjacent regularities occur. Newport and Aslin examined adults’ detection of conditional relations among non-adjacent elements that did and did not adhere to this natural language structure: non-adjacent consonants (with one unrelated intervening vocalic segment), non-adjacent vowels (with one unrelated intervening consonantal segment), and non-adjacent syllables (with one intervening syllable that was unrelated). In accord with the structure of natural languages, adults seemed to be unable to track the relations between non-adjacent syllables, where the intervening element was of the same kind (a syllable). Even when the patterns were quite simple and participants were given extensive exposure to the patterns (in one case over 10 days of repeated exposures), participants remained unable to track relations between non-adjacent syllables. In contrast, adults readily learned the relations between non-adjacent consonants and vowels, where the intervening element was a different kind from that in which the non-adjacent regularities occurred. These findings suggest that constraints on statistical learning may help to explain the universal aspects of these patterns in natural languages. Similar to Conway and colleagues’ results (Conway and Christiansen, 2009; Emberson et al., 2011), these findings also demonstrate that human learners preferentially track statistical information only in particular types of environmental input. Such findings highlight the importance of considering statistical learning in its broader environmental context, including the nature of the input to which the learner is exposed, as well as the cognitive, developmental, and attentional state of the learner.

### The state of the learner

Human learners are characterized by perceptual biases and cognitive constraints. Appreciating the influences of learners’ biases and developmental state on statistical learning is necessary for a complete understanding of the extent and limits of this domain-general learning process across development.

#### Spatiotemporal biases and perceptual similarity

Consideration of learners’ perceptual biases is especially important for understanding constraints on visual statistical learning, as such biases have been shown to influence the types of statistics learners extract from visual scenes (Fiser et al., 2007). One general perceptual bias exhibited by infants and adults is the bias to perceive objects as moving along specific trajectories given certain visual and/or auditory cues (e.g., Sekuler and Sekuler, 1999; Shimojo et al., 2001). When observing two identical objects moving toward each other, coinciding, then moving away from each other, two interpretations are possible: (1) the two objects streamed past one another (*streaming*), or (2), the two objects bounced off of one another (*bouncing*). Various perceptual features such as the acceleration of the objects (Sekuler and Sekuler, 1999; Fiser et al., 2007) or the presence of a sound at the time of coincidence (Sekuler et al., 1997; Watanabe and Shimojo, 2001) bias observers toward one of these two interpretations.

Fiser et al. (2007) investigated whether this perceptual bias to perceive objects as moving along specific trajectories affected the types of statistics adult learners computed from visual events. Participants observed a single object move behind an occluder and then saw two objects emerge from behind the occluder simultaneously. One object emerged from the occluder following the same trajectory as the first object. The second object emerged from the occluder at a 90° angle to the original trajectory. Thus, presentations could be interpreted two different ways: (1) as an object streaming behind the occluder on a straight trajectory, or (2) as an object bouncing off of a surface behind the occluder and reemerging on the same side that it originated.

To examine whether perceived motion trajectories would bias statistical learning, Fiser et al. (2007) manipulated the acceleration of the objects to bias observers toward one of these two percepts. Objects moving at constant speed produced a streaming percept whereas decelerating-accelerating objects produced a bouncing percept. If visual statistical learning mechanisms compute all available temporal co-occurrences of shape pairs, then learners should acquire transitions from the first shape to each of the two later shapes equally well, regardless of whether observers were biased toward streaming or bouncing percepts. However, this is not what Fiser et al. found. Rather, adults preferentially learned the associations consistent with the perceptual bias of streaming or bouncing they had during familiarization. Thus, this perceptual bias constrained statistical learning to shape pairs consistent with that bias.

The influence of perceptual biases on statistical computations is not limited to statistics in visual scenes. Similar to spatiotemporal biases, Gestalt principles of perception have been shown to constrain the detection of statistical relations in both auditory and visual input (Baker et al., 2004; Creel et al., 2004; Newport and Aslin, 2004; Emberson et al., 2011). For example, Creel et al. (2004) demonstrated that Gestalt principles of element similarity interact with temporal adjacency in determining what kinds of auditory statistical regularities are learned. In this experiment, adult participants were presented with two interleaved streams of tone triplets such that participants heard the first tone of the first triplet stream, followed by the first tone of the second triplet stream, then the second tone of the first stream, then the second tone of the second stream, and so on (Creel et al., 2004). The result of this interleaving was that triplets could only be detected via sensitivity to non-adjacent conditional relations.

Interestingly, adults showed no learning of the tone triplets, only sensitivity to the less reliable relations between adjacent elements in the stream. However, when Creel et al. (2004) included perceptual grouping cues, by presenting the two interleaved streams in differing pitch ranges or timbres, adults became sensitive to the conditional relations between the similar, yet temporally non-adjacent, elements. This finding suggests that Gestalt principles of similarity interact with temporal adjacency in constraining statistical learning.

#### Availability of cognitive resources

Thus far, our discussion has highlighted similarities in infants’ and adults’ sensitivities to statistical information. Researchers hold differing views, however, on how implicit statistical learning abilities may change across development (e.g., Thomas et al., 2004; Janacsek et al., 2012) or remain constant across development (e.g., Reber, 1993; Vinter and Perruchet, 2000).

In some studies reporting developmental differences, older individuals show better learning than younger individuals (e.g., Maybery et al., 1995). Consistent with this possibility, infants provide evidence for tracking increasingly complex statistical regularities in visual sequences with age: 2- 5- and 8-month-old infants distinguished structured from random sequences composed of six looming shapes (Kirkham et al., 2002), but newborn infants only distinguished structured from random sequences when the sequences contained four, not six, items (Bulf et al., 2011).

In other cases, however, younger individuals outperform older individuals (e.g., Jost et al., 2011; Janacsek et al., 2012). Jost et al. (2011) compared the time course of children’s and adults’ implicit learning by examining participants’ ERPs during a visual statistical learning task. Participants observed a series of stimuli presented one at a time on a screen and pressed a button whenever the target stimulus appeared, which was predicted at different levels of probability by the stimuli immediately preceding the target. Jost et al. found that children exhibited learning-related ERP components earlier in the study than adults, suggesting that children required less exposure to the patterns to detect the statistical structure.

To explain differences in statistical learning ability across development, researchers have appealed to domain-general, maturational constraints on perception and memory. Bulf et al. (2011) suggested that newborns’ limited attentional and working memory capacities may inhibit statistical learning efficiency. Interestingly, researchers have posited a similar explanation to account for findings of children outperforming adults. In that case, however, researchers have offered the paradoxical idea that maturational constraints on perception and memory confer a computational advantage for some types of learning (e.g., Newport, 1988, 1990; Elman, 1993). In particular, Newport’s (1990) “Less is More” hypothesis assumes that children’s abilities to perceive and store complex stimuli is reduced compared to those of adults, and suggests that such limitations give children an advantage for tasks requiring componential analysis because children are better able to identify and process component parts. Adults, in contrast, attempt to perceive and store stimulus relations of greater complexity.

Suggestions that maturational constraints on perception and memory can both hurt and help performance in tasks requiring componential analysis appear contradictory. However, most empirical support for Newport’s “Less is More” hypothesis (1990; e.g., Kersten and Earles, 2001) comes from child and adult populations, leaving open the possibility that very early increases in infants’ relatively limited perception and memory abilities may be positively related to statistical learning ability. To our knowledge, however, Bulf et al.’s (2011) hypothesis that limited cognitive resources limit newborns’ statistical learning performance has not yet been confirmed independently. Although visual working memory performance increases roughly linearly across the first postnatal year (Diamond, 1985; see Bell and Morasch, 2007 for a review), a number of other early developments could, in principle, be responsible for changes in statistical learning (e.g., different spatiotemporal biases due to changes in perceptual acuity). An important avenue for future research will be to investigate these possibilities, beginning by examining the relation between the development of infant working memory ability and statistical learning ability.

In addition to maturational constraints on perception and memory, the allocation of attentional resources may also play a role in constraining statistical learning. Although some researchers have argued that statistical learning is an “automatic” (i.e., implicit, rapid) process (e.g. Saffran et al., 1997), other researchers have found reason to suggest that statistical learning both is and is not automatic (e.g., Turk-Browne et al., 2005). It is automatic in that statistical computations seem to be carried out without conscious intent and often without awareness that any structure was learned (e.g., Saffran et al., 1997; Meulemans et al., 1998; Turk-Browne et al., 2005). However, statistical learning is not automatic in that it operates better over attended versus unattended input (e.g., Toro et al., 2005; Turk-Browne et al., 2005; Emberson et al., 2011). For instance, when two interleaved streams of shapes are presented to observers in two different colors, and participants are instructed to attend to only one color, only the statistical relations in the attended color are learned (Turk-Browne et al., 2005). This attentional constraint on statistical learning appears to be one of its most general limitations, likely constraining detection of statistical regularities regardless of input domain or modality (e.g., Emberson et al., 2011).

#### Prior experience

In addition to maturational changes in cognitive resources, such as working memory capacity and attention, another important aspect of development is learning from experience interacting with the environment. Expectations about the structure of the environment undergo rapid changes in the first years after birth due to experiences interacting with the world (e.g., Campos et al., 1992; Adolph et al., 1993). Such changes in learners’ expectations about the structure of their environment may have the potential to influence statistical learning processes (Thiessen, 2010). For example, years of experience with language may provide adults with strong expectations that words and objects relate to one another (e.g., Namy and Waxman, 1998).

Thiessen (2010) investigated how such expectations influence adults’ statistical learning of word-object associations. Adults were presented with paired audio-visual information in which word boundaries as well as word-object associations were statistically defined. Participants tracked both of these statistical relations simultaneously, and word segmentation benefited from the addition of word-object associations. When adults were presented with tonal rather than linguistic stimuli, however, they did not benefit from the regular relations between tone words and objects. Thiessen suggested that experience with language may predispose adults to expect words and objects to relate to one other, such that they are sensitive to these associations in linguistic input, but not in tonal input. This hypothesis leads to the prediction that young infants may not benefit from word-object relations even with linguistic input, because they may not yet have built up the expectation that words relate to objects (e.g., Werker et al., 1998). This is precisely what Thiessen found; similar to adults in the tonal condition, 8-month-old infants’ ability to segment words did not benefit from the presence of word-object relations, regardless of whether linguistic or non-linguistic input was used.

Thiessen’s (2010) findings demonstrate the role of prior experience and learners’ expectations in facilitating computation of previously ignored statistics. Other research, however, indicates that prior experience can impede statistical computations. For example, Gebhart et al. (2009) presented adult learners with auditory sequences of trisyllabic nonsense words defined by the TPs between syllables. When the researchers altered the organization of the nonsense words mid-way through the familiarization stream, participants only learned the first of the two structures. Participants detected words in both structures only when exposure to the second structure was tripled in duration, or when the transition between structures was explicitly marked. Thus, successful extraction of the statistical regularities in one auditory structure inhibited learning of a subsequent auditory structure.

## Mechanisms Underlying Statistical Learning

How is it that statistical learning can be so constrained while still adapting flexibility to input across domains and modalities? The reason for both flexibility and constraints on statistical learning is likely because the environment contains both variance and invariance; organisms need a way to flexibly adapt and generalize to different contexts while simultaneously honing in on the types of structures that are most consistent and informative in the environment. What is less clear are the mechanisms by which statistical learning occurs and how these mechanisms are configured to allow for both flexibility and constraints.

We began this review by introducing statistical learning as sensitivity to transitional probabilities (TPs), and this view was predominant in the early days of infant statistical learning research that focused predominantly on word segmentation. However, there is now a wealth of data on infants’ and adults’ statistical learning across domains, and this calls for a broader view of statistical learning (e.g., Saffran, 2001; Maye et al., 2002; Thiessen and Saffran, 2003; Graf Estes et al., 2007; Smith and Yu, 2008; Frank et al., 2010). For example, consider Saffran’s (2001) and Graf Estes et al.’s (2007) findings that the output of statistical learning is entire word-like units, not simply highly probable sound sequences. A mechanism that only tracks probabilistic relations between elements cannot fully account for such a finding (see Thiessen et al., 2012). Moreover, even in segmentation tasks, models designed to track transitional probabilities do not always accord well with human performance (see Frank et al., 2010).

A variety of alternate models of statistical learning have been proposed that do not rely on explicitly computed statistics. It is not yet clear which type of model produces the most valid account of human learning processes across tasks (Frank et al., 2010). A complete review of all such models is beyond the scope of this review; instead, we briefly describe one well-known model, PARSER (Perruchet and Vinter, 1998), to illustrate that there are multiple possible mechanisms to account for statistical learning data.

PARSER (Perruchet and Vinter, 1998) is a type of “chunking” model that produces the same segmentation results as Saffran et al. (1996a,b) by implementing basic laws of attention, memory, and associative learning, rather than by computing statistics such as transitional probabilities. PARSER is modeled on the principle that perception guides internal representation. Briefly, units that are perceived within one attentional focus are “chunked” into a new representational unit. The fate of these new representations depends on fundamental principles of memory: internal representations of chunks that are repeated are progressively strengthened, and representations of chunks that are not repeated are forgotten (Perruchet and Vinter, 1998). Applied to Saffran et al.’s (1996a,b) segmentation task, PARSER would first randomly segment the speech stream into small chunks. Because chunks have a greater chance of being repeated if they are part of the same word than if they span a word boundary, internal representations of words or parts of words will be stronger in memory than representations of non-words and chunks spanning word boundaries. Thus, PARSER can account for Saffran et al.’s (1996a,b) findings of participants’ greater sense of familiarity for words than non-words or part-words.

As noted, several models of statistical learning employing quite different mechanisms have been proposed to account for the various findings of the statistical learning literature, but no model has yet been proposed that can account well for human performance across statistical learning tasks (Thiessen et al., 2012). In particular, what is lacking are models that achieve sensitivity to other statistical relations in addition to conditional relations, such as the central tendency of a set of elements (distributional statistical learning; e.g., Maye et al., 2002), as well as models that account for human’s learning and generalization based upon similarity across items extracted from the input (e.g., Thiessen and Saffran, 2003). Thiessen et al. (2012) argued that mechanisms designed only to account for the extraction of units, such as segmenting words from a speech stream, cannot account for these other forms of statistical learning.

Thiessen et al. (2012) proposed a framework that attempts to account for these various forms of statistical learning by combining processes of extraction with processes of comparison across extracted segments in an iterative model whereby the discovery of new structures via comparison serves to educate the extraction processes. To illustrate this idea, consider the finding that when syllable stress and statistical cues indicated different word boundaries in a speech stream, 7-month-olds segmented based on statistical cues, whereas 9-month-olds segmented based on stress cues (Thiessen and Saffran, 2003). Models that are only designed to account for segmentation cannot explain these findings without positing additional changing constraints on the learner or on the statistical learning mechanism itself. In contrast, Thiessen et al.’s (2012) framework accounts for such findings without necessitating new or changing constraints; according to this framework, such findings demonstrate initial segmentation based on conditional statistics followed by comparison across segmented words, allowing the discovery of patterns of stress cues in English words, which in turn inform the process of segmentation in the future.

Although Thiessen et al.’s (2012) framework has not yet been implemented into a working computational model, such a framework pushes the field forward by offering a mechanism that accounts for developmental differences in statistical learning. Moreover, this framework is also helpful for thinking about the origins of the constraints on and flexibility of statistical learning. That is, the framework is based on general processes of attention, memory, and comparison that likely govern extraction and generalization across domains. Furthermore, this framework describes a way in which learners may use a constrained, limited-capacity mechanism to flexibly adapt to different characteristics of the input over time.

## Conclusion

Statistical learning is a means of uncovering structure in complex environmental input. It operates in both auditory and visual domains, and encodes multiple types of statistics simultaneously. Constraints on statistical learning serve to reduce the number of possible associations available, making statistical learning tractable.

A comprehensive model of statistical learning across domains has not yet been reported in the literature, but much progress has been made in uncovering the origins of both the flexibility of and constraints on statistical learning. Specifically, flexibility may be the result of mechanisms built upon domain-general processes, such as attention, memory, and perception, rather than domain- or modality-specific processes. Flexibility may be built into the system as a product of learners’ ability to discover new structures via comparison, and use those new structures to influence further extraction (Thiessen et al., 2012). Constraints on statistical learning are driven by a variety of factors: limited attention, perception, and memory capacity, as well as maturational increases in these domain-general processes; learned biases and expectations about the structure of the environment; and ways in which statistical tendencies in language have been shaped to fit the human brain, rather than vice versa.

Thus, while research has revealed numerous influences on the various constraints on statistical learning, the principal contribution to flexibility in statistical learning appears to be its domain-general nature. Nevertheless, the domain-generality of statistical learning mechanisms has been hotly debated. Some researchers interpret demonstrations of statistical learning across domains and modalities as evidence of a single, domain-general statistical learning mechanism (e.g., Kirkham et al., 2002), but others contend that statistical learning cannot be domain-general due to observed modality-specific constraints (Conway and Christiansen, 2005, 2009; Emberson et al., 2011). Specifically, they cite findings such as the auditory-temporal, visual-spatial distinction as evidence for separate statistical learning mechanisms for each modality (Conway and Christiansen, 2009). One limitation of this line of reasoning, however, is that constraints differentially affecting statistical learning of different types of input *within* modalities (e.g., Endress, 2010; Thiessen, 2010) would necessitate multiple statistical learning mechanisms *within* modalities as well as across modalities. Thus, the domain-general view seems to be the most parsimonious account of the data. However, evidence supporting a domain-general account of statistical learning does not exclude the possibility of multiple domain- or modality-specific statistical learning subsystems. Further research is needed to determine which of these views provides the most complete account of statistical learning. Research examining statistical learning performance using comparable tasks across domains and modalities, as well as research comparing the ability of modality-specific and domain-general computational models to fit such human data, may be particularly informative.

Moreover, future research should continue to investigate the type of flexibility in statistical learning documented by Turk-Browne and Scholl (2009), who demonstrated flexibility in the transferability of the representations that emerged from adults’ visual statistical learning. Further research should pursue similar lines of research employing other tasks and input types to investigate the generalizability of such findings across modalities. A final important avenue for future research will be to continue working toward developing a comprehensive model that can accommodate the various forms of statistical learning (sensitivity to conditional relations, distributional statistics) across domains as well as developmental changes in such learning. Longitudinal research and research that makes within-subjects comparisons across tasks may be particularly useful in this endeavor.

## Conflict of Interest Statement

The authors declare that the research was conducted in the absence of any commercial or financial relationships that could be construed as a potential conflict of interest.
